# Structural barriers to implementing individual placement and support in Japanese disability policies and Labor systems

**DOI:** 10.3389/phrs.2026.1608934

**Published:** 2026-07-07

**Authors:** Sosei Yamaguchi, Teruo Hayashi

**Affiliations:** 1 Department of Community Mental Health & Law, National Institute of Mental Health, National Center of Neurology and Psychiatry, Kodaira, Japan; 2 Seiwakai Nishikawa Hospital, Hamada, Japan

**Keywords:** employment service policy, implementation and dissemination, individual placement and support, Japanese system, vocational services

## Abstract

**Background:**

This study aimed to contextualize structural barriers to the implementation of Individual Placement and Support (IPS) in Japan.

**Analysis:**

We examined the position of Japan’s employment service policies for individuals with mental illness within the OECD framework for IPS implementation and identified key challenges. A mixed-methods approach was employed, combining a narrative search of Japanese policy documents with systematic searches of Japanese IPS research. According to the OECD framework, Japan’s IPS implementation was positioned between the stages of “Building the foundations for integrated mental health, skills, and work policy” and “Shifting from trials to a scaled-up integrated approach.” A major barrier was the absence of a comprehensive vocational service system for individuals with a diagnosis of severe mental illness.

**Policy Options:**

Advancing IPS dissemination in Japan requires multifaceted strategies, including grassroots efforts by service providers, strengthened advocacy, incorporation of employer and client perspectives, and consideration of resource allocation across populations with varying levels of needs.

**Conclusion:**

While macro-level research on IPS implementation remains scarce in Japan, the practical and feasible policy considerations identified in this study could be beneficial in Japan as well as other non-Western contexts encountering similar implementation challenges.

## Background

People with a diagnosis of severe mental illness (SMI) face challenges in obtaining competitive employment in Japan. Globally, approximately 60% of individuals in this population are interested in securing competitive employment [1], and a Japanese study found a similar percentage among people with mental illness in sheltered workshops [2]. Over the past two decades, Japan has been striving to support the employment of people with disabilities. Nevertheless, only an estimated 220,000 individuals with mental illness worked at companies with five or more employees in 2023 [3], representing only a small proportion of the approximately 1,450,000 people holding a mental disability certificate [4]. These facts indicate that nationwide implementation of effective employment services for people with a diagnosis of SMI remains an issue.

The individual placement and support (IPS) model was developed in the United States to address unemployment among people with a diagnosis of SMI. IPS is well-grounded in eight principles (e.g., zero exclusion criteria, rapid job search, client preferences) [5]. A recent comprehensive review recognized the effectiveness of IPS in terms of vocational outcomes globally [6], and systematic reviews for economic evaluation also support its cost-effectiveness [7, 8]. In addition, an IPS fidelity scale was developed to maintain service quality [9]. This evidence has led to IPS being incorporated into national and state policies in Western culture countries such as the United States, the United Kingdom, the Netherlands, Norway, Australia, and New Zealand [5].

**FIGURE 1:**
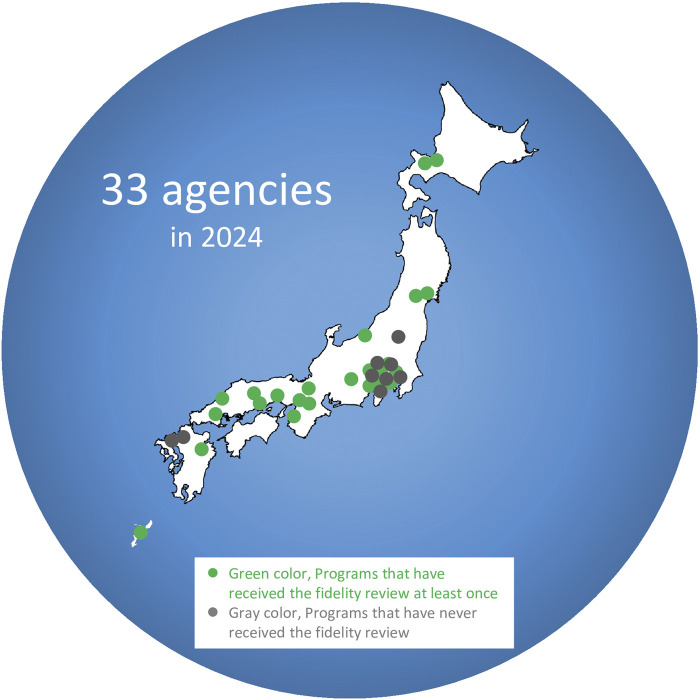
Geographic distribution of agencies implementing individual placement and support programs across Japan (Japan. 2025).

Several studies on IPS have also been conducted in Japan. A scoping review showed that both the effectiveness and the cost-effectiveness of IPS were superior to usual vocational services, based on randomized controlled trials in Japanese settings [10]. Moreover, fidelity research has produced rich evidence on replication of IPS service contents and short- and long-term positive vocational outcomes in routine settings [11–16]. The accumulated evidence has led to Japan’s IPS progressing from the experimental stage to the implementation and dissemination stage. As of April 2025, more than 30 agencies have implemented IPS programs (Figure 1).

Despite clear evidence and successful implementation, broader dissemination of IPS in Japan remains limited compared to Western countries. Hayashi et al. [10] estimated that IPS agencies account for less than 1% of employment service agencies overall. To address this situation, an understanding of the macro-environment, such as employment service policies and finding, is essential. For example, the OECD has proposed a framework for implementing evidence-based employment services from a systems perspective. This framework comprises four stages: 1) developing the right rhetoric; 2) building the foundations for integrated mental health, skills, and work policy; 3) shifting from trials to a scaled-up integrated approach; and 4) integrated mental health, skills, and work plans in practice (Supplementary Table 1) [17]. Similarly, a detailed review from New Zealand synthesized system-level challenges from a broad perspective, including health, welfare, and vocational rehabilitation policies, to inform the scale-up of evidence-based services [18]. However, little is known about structural barriers of IPS related to Japan’s unique mental health systems and disability policies. In the current study, we aim to contextually analyze such barriers to IPS and thus contribute to system consideration, particularly in non-Western countries.

## Analysis

### Analytical methodology

We first positioned Japan’s employment service policy for people with mental illness within the OECD framework for implementing IPS [17]. To incorporate this framework into the Japanese setting, we conducted a narrative search for policy information on employment services for people with mental illness issued by the Ministry of Health, Labor and Welfare (MHLW), using the MHLW website and Google. Additionally, a systematic search for MHLW-funded IPS research reports was conducted using the query “(IPS OR supported employment) AND (mental OR schizo*).” We searched PubMed and the Japanese database Ichushi for peer-reviewed articles on the policy context of IPS in Japan, using the following title/abstract keywords: “(mental OR schizo*) AND (IPS OR supported employment) AND (Japan*).” We then positioned Japan within the OECD framework to identify its current implementation stage and key system-level challenges and to consider potential policy options for supporting wider dissemination of IPS.

### Current policy information on employment services for people with mental illness

Within Japan’s health and welfare system, three divisions of the MHLW are involved in employment-related services, namely, the Labor Division, the Disability Welfare Division, and the Mental Health and Disability Division (Table 1; Supplementary Table 2). While these divisions cooperate with each other, they have their own missions, and their employment services are administered under separate budgets. In addition, among the 32 OECD countries, Japan ranked as one of the countries with the low total expenditure on employment support [19]. Furthermore, the MHLW recognized IPS as effective employment model for people with a diagnosis of SMI in the United States and United Kingdom [20].

**TABLE 1:** Characteristics of employment services for obtaining a competitive job in the three divisions (Japan. 2025)

Division	Labor division (labor bureau)	Disability welfare division	Mental health and dsability division
**Type of service**	Work and life support centers for persons with disabilities	Transition support for employment	Psychiatric day-care
**Background law**	Act for promotion of employment of persons with disabilities	Comprehensive support act for persons with disabilities	Health insurance law
**Main service and model**	• Support to find a job • Coordination with other employment services	• Support to find a job • Group-based services in agency • Train-then-place model focusing on work-readiness	• Rehabilitation • Group-based services in agency • Train-then-place model focusing on work-readiness
**Client**	Individuals with any disabilities	Individuals with any disabilities	Individuals with mental illness
**Staff assignment**	• Employment specialist • Living support staff	• Manager • Employment specialist • Job trainer • Living support staff	• Doctor • Either nurses or occupational therapists • Either social workers or psychologists
**Caseloads**	No limit	15 cases per full-time employment specialist 6 cases per job trainer	No limit
**Statutory maximum utilization period**	No limit	2 years	No limit
**Number of agencies**	337 agencies in 2024	3,301 agencies in 2023	Around 1,537 agencies in 2016
**Payment system**	Annual grants are paid to each agency	• Each agency is reimbursed on a per-service basis. • If the agency employment rate exceeds 50%, the amount of reimbursement is doubled. • No reimbursement is provided for follow-up services after employment.	Each agency is reimbursed on a per-service basis.
**Sector**	Most agencies are managed by private social welfare corporations.	Most agencies are managed by private corporations or non-profit organizations.	Most agencies are managed by private psychiatric hospitals.

#### Labor division (labor bureau)

The Labor Division is responsible for promoting disability employment measures aimed at realizing an inclusive society in which persons with disabilities can fully participate in work. It has central role in protecting the work environment, including disability employment in Japan. In particular, the Act for Promotion of Employment of Persons with Disabilities provides direct employment services, mandates employment rates for people with disabilities, and stipulates certain subsidies for companies that employ individuals with disabilities. Direct employment services are provided by 337 disability employment and life support centers across Japan that assist people with all types of disabilities [21]. Most of these centers lack mental health professionals and have notably high caseloads per staff member, owing to no caseload limits. A cross-sectional survey of 168 agencies revealed that, on average, each agency employed four staff members and served approximately 380 clients [22]. Consequently, employment services given to people with mental illness are very limited.

**FIGURE 2:**
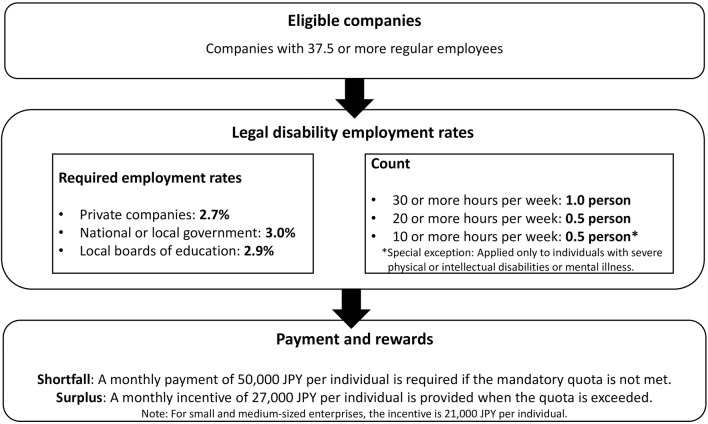
Overview of the system for the mandated disability employment rate under the Act for the Promotion of Employment of Persons with Disabilities (Japan. 2026).

**FIGURE 3:**
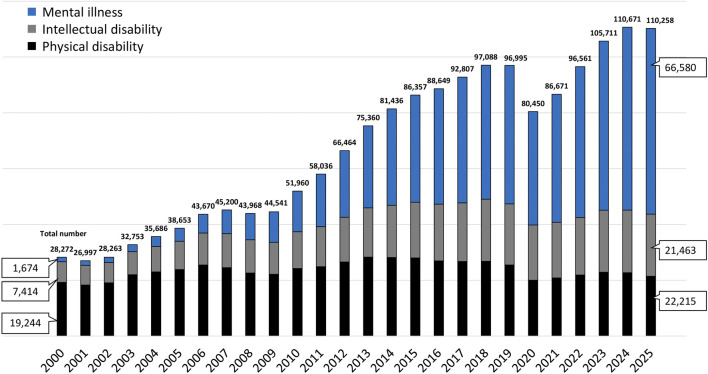
The annual number of individuals with disabilities obtaining new employment through the Public Employment Security Offices (Japan. 2025).

The law also mandates designated job offers for people with disability. It further stipulates disability employment rates, requiring companies with 37.5 or more regular employees to ensure that individuals with disabilities constitute 2.7%–3.0% of their total workforce (Figure 2). This legal obligation has provided a powerful boost to the employment of people with disabilities. In 2023, around 55,000 companies employed a total of approximately 130,000 individuals with mental illness, using the designated offers under this law [23]. Indeed, the number of people with mental illness who found new employment via public employment security offices has increased from approximately 1,600 in 2000 to 65,000 in 2025 (Figure 3; Supplementary Table 3) [24]. Nevertheless, three key concerns have consistently been raised. First, most jobs designated to fulfill the mandated disability employment rates tend to be unstable and have low salaries. Second, the designated job offers include fewer occupation types than other job offers. Third, in calculating the disability employment rate, companies can still meet the required quota by placing employees with disabilities in a single department or by outsourcing such positions to other companies. Although the majority of designated job vacancies are for integrated workplaces, this third issue has recently drawn particular criticism [25], as such employment does not necessarily align with the principle of social inclusion.

#### Disability welfare division

The Disability Welfare Division is mandated to promote disability welfare policies that enable persons with disabilities to live and participate as full members of their communities. It establishes a wide variety of assistance systems for people with disabilities, such as housing services, day services, counseling services, sheltered workshops, and vocational services. In particular, Transition Support for Employment (TSE) within the Comprehensive Support Act for Persons with Disabilities provides vocational services to individuals to find and maintain a new job. TSE is designed for people with disabilities under 65 who wish to work and are deemed capable of employment, but there are no recommended measures for assessing vocational abilities among those seeking to use TSE. Over 3,000 agencies have addressed TSE across Japan and play a central role in facilitating employment for people with disabilities [26]. Most TSE agencies have been administered by non-profit organizations or private corporations, including commercial enterprises.

The reimbursement system for TSE is unique. Each agency offering TSE is reimbursed on a per-service basis (i.e., how many times each client visits the agency office), regardless of service type (e.g., outreach services, individual services, group-based services). When the agency achieves an employment rate of 50% or higher among its clients, the reimbursement from national and local governments doubles (around JPY 6,000 [40$] to JPY 12,000 [80$] per service). This reimbursement incentive allows each agency to count the number of employment cases without considering the severity of symptoms and disabilities. While TSE also aims to support job retention after employment, agencies gain no reimbursement for follow-up services within the first 6 months after employment. From a managerial perspective, the most profitable model implies that (1) staff members help individuals with mild disabilities, such as those who can attend the agency three or four times a week, and (2) provide in-house group-based services for increasing work-readiness to many clients at the same time.

#### Mental health and disability division

Since 2017, the Mental Health and Disability Division has advanced its mission to promote a community-based integrated care system in which all individuals, regardless of the presence or severity of mental health conditions, can live safely in their communities with comprehensive access to medical and social services, including employment support. The Division covers inpatient and outpatient psychiatric service systems, as well as psychiatric rehabilitation and community service systems. Specifically, psychiatric day-care centers often have a multi-disciplinary team addressing vocational rehabilitation. On average, each center has approximately 70 registered clients [27]. However, psychiatric day-care is currently designed to provide group-based services within the agency space and does not allow staff members to provide outreach services. Each psychiatric day-care is reimbursed on a per-service basis. In addition, while most psychiatric day-care centers in Japan have been administered by private psychiatric hospitals [27], clients’ employment means reduced use of day-care and less revenue for the hospitals.

### IPS projects and policy proposals

Through a systematic search of MHLW-funded IPS research reports, we identified four IPS projects and corresponding reports (Supplementary Figure 1). The main findings of these projects have been published in peer-reviewed journals. These consisted of relatively small-scale trials examining the effectiveness or cost-effectiveness of IPS [28, 29], fidelity surveys [11], and service process analyses [12]; however, none of them included a macro-level policy analysis. In addition, these IPS projects were conducted by a single health research department, potentially limiting the diversity of stakeholder perspectives on IPS. Furthermore, we identified only two peer-reviewed academic papers that addressed policy proposals related to IPS. These studies emphasized the need for integrated vocational and mental health services and highlighted that existing (reimbursement) systems providing in-house group-based services are not well aligned with IPS or with employment services for individuals with a diagnosis of SMI who require outreach support [10, 30]. As a result, TSE and psychiatric day-care agencies in Japan tend to focus on providing in-house group training to improve work readiness as well as the cognitive and vocational functions of individuals with mental illness [30].

### OECD framework and major issue

Based on the OECD framework, Japan’s system for implementing IPS appears to fall between Stage 2 (*building the foundations for integrated mental health, skills, and work policy*) and Stage 3 (*shifting from trials to a scaled-up integrated approach*). In particular, the Mental Health and Disability Division recognizes the importance of providing integrated services for all individuals, regardless of the presence or severity of mental health conditions. Although IPS programs have gradually expanded following initial research efforts, they have not been scaled up.

The most substantial problem for the implementation of IPS appears to be the lack of a vocational service system for people with a diagnosis of SMI such as schizophrenia. These individuals are the primary target of IPS who often need outreach support. Whereas the number of employed individuals with mental illness largely increased in Japan [31], the proportion of those with a diagnosis of SMI remains small. For example, among the estimated 220,000 people with mental illness who were employed in 2023 by companies with five or more employees, only around 10% had a diagnosis of schizophrenia [3]. In another example, recent Japanese studies reported that a collaborative program between psychiatric day-care and public employment security offices has achieved high employment rates (around 75%). However, the clients’ mean scores of global assessments of functioning (GAF) were approximately 70 at baseline [32, 33] in contrast to the mean GAF scores of around 50 in Japanese IPS studies [12, 14, 29]. These facts imply that people with a diagnosis of SMI or low functions have been potentially excluded from the existing employment systems and services. Additionally, as already mentioned, since most TSE agencies and psychiatric day-care are administered by private organizations, a “financially effective business model” is more likely to benefit individuals who can attend in-house training several times a week for a long period of time. In other words, implementing an IPS model that delivers employment support for people with a diagnosis of SMI and enables them to quickly find a job is difficult under the current systems. Although the Japanese employment service system does not have formal eligibility criteria for potential clients to access specific services, collecting data on rejection rates in real-world settings should be a priority for future research.

## Policy options

The OECD framework recommends a phased implementation strategy and appropriate resource allocation for evidence-based employment services [17]. To progress through these stages, previous studies have suggested several approaches, including cross-sectoral collaboration and shared goal setting, the establishment of learning communities, advocacy activities, and active engagement of policymakers [17, 18, 34–36]. Among these options, we need to consider practical and feasible approaches in Japan. Our review indicates that a central challenge lies in reconstructing the employment service system to effectively deliver IPS to individuals with SMI, who have substantial support needs, while also being consistent with the IPS principle of “zero exclusion” [5]. Targeting individuals with a diagnosis of SMI would automatically force the employment services to focus on providing adequate outreach and follow-up services—core elements of IPS—based on individual preferences rather than training-based and non-evidence-based employment services. In this context, the first step for disseminating IPS involves integrating these core elements into the existing employment service system, rather than extensively overhauling the existing disability employment system. The current study proposes three approaches to address intertwined structural barriers and highlights one necessary discussion.

### Increasing the number of IPS providers through grassroots activities

The first approach for addressing the structural barriers to IPS involves gradually expanding IPS agencies and promoting IPS awareness among policymakers through grassroots-level activities, leveraging the support of professional groups such as the Japanese Individual Placement and Support Association (JIPSA)^1^. Established in 2016, JIPSA is a voluntary organization comprising practitioners who provide IPS services beyond the existing system. It plays roles in creating a community of practitioners who want to learn IPS, in negotiating with policymakers, and in building evidence through research. Historically, Japan’s welfare policy environment, unlike that of other developed countries, has not emphasized or utilized scientific evidence for policymaking [37]. In other words, services tend to be instituted based on customs and practices that are already widely accepted by service providers. Consequently, a viable and practical strategy for diffusing IPS will entail increasing the number of stakeholders who empathize with IPS principles and developing financial incentives, particularly tailored for outreach and follow-up services, rather than pursuing entirely new policies precisely aligned with IPS. Under the current Japanese system, rapid expansion or replication of exemplary IPS programs poses financial challenges for individual agencies, even with JIPSA’s support. In this context, acknowledging service providers who are interested in IPS is essential, despite their services not having complete IPS fidelity, because these so-called “quasi-IPS” programs could potentially grow into “mature IPS” programs with the support of existing IPS communities such as JIPSA. Another approach would be to encourage testing IPS with a small number of clients who want to start work early, as it would not significantly affect the agency operations. Such flexible grassroots activities to support IPS would act as a catalyst for policy reorientation towards evidence-based employment services.

### Advocacy activities

Another potential strategy for addressing the structural barriers to IPS is based on human rights for people with mental illness. In September 2022, the United Nations published the results of a review of Japan under the Convention on the Rights of Persons with Disabilities (CRPD) [38]. This report criticized the Japanese employment service system, including such elements as the sheltered workshops with very low salaries and unintegrated workplaces, and the Japanese government has consequently sought ways to resolve these issues. IPS may be viewed as a valuable tool by policymakers because one of its strengths pertains to facilitating inclusive workplaces through helping individuals work in competitive employment settings. Indeed, IPS has been rapidly disseminated across European countries, as employment issues for people with mental illness are considered a human rights issue relating to CRPD in these countries [39]. Drawing from lessons learned in a European context, a discussion based on the broader perspective of human rights rather than simply employment services may contribute to the dissemination of IPS in Japan.

### Employers’ and clients’ perspectives

Engaging diverse stakeholders is essential for wider IPS implementation. Although employer perspectives on IPS have rarely been studied in Japan, mandated disability employment quotas may increase employer awareness. Notably, the National Association of Vocational Foster endorsed IPS and, in collaboration with JIPSA, produced educational films in 2025^2^. Since 2024, Japanese employers have been legally required to provide reasonable workplace accommodations. However, a recent survey by an advocacy group led by people with lived experience of mental illness indicated that many individuals with similar experience do not receive appropriate accommodations [40]. The group recommended promoting IPS to provide continued support after job placement. Arranging partnerships with employers and advocacy groups may accelerate IPS implementation.

### Balancing service costs between people with mild and severe mental illness

Potential challenges in disseminating IPS throughout Japan include ensuring constructive policy discussion on how to appropriately allocate budgets according to the severity of the disability and thus maximize employment support services and cost-effectiveness. Although IPS has been demonstrated to yield desirable and cost-effectiveness outcomes [28, 29], particularly for individuals with a diagnosis of SMI, those with milder conditions may not require such intensive support. Since current Japanese policies have successfully increased the employment of individuals with disabilities overall, policymakers may worry that allocating service resources to IPS may lead to a decline in employment opportunities for this population. Ideally, policy design supported by ample budgets should ensure that each client receives support commensurate with their needs. In this context, one possible proposal would be to make minimal changes to the policy to enable quasi-IPS services to support a small number of users within TSE agencies. Another proposal would be targeted investment for individuals with the greatest needs, reflecting a more equitable redistribution of resources. However, reliably assessing service needs presents substantial challenges. Furthermore, eligibility and severity assessments could raise potential ethical concerns if individuals are excluded from IPS on the basis of assessment results. While there is no definitive solution for these issues, they will inevitably be part of the broader policy discussions on the national expansion of IPS in Japan. To support a fair and evidence-informed discussion, national data will likely be required to estimate the costs and return on investment of IPS implementation in the future. Furthermore, if assessments are conducted during the implementation of an IPS, it is essential to ensure that evaluation criteria centered on individual preferences and aspirations are incorporated into the structure, thus guaranteeing the principles of the IPS.

## Conclusion

Despite accumulating evidence on IPS effectiveness, structural and policy-related barriers limit its dissemination in Japan. These challenges stem from a fragmented employment system that often prioritizes individuals with milder disabilities and a privatized service landscape favoring group-based, in-house training over core IPS elements such as individualized outreach services. A pragmatic dissemination strategy involves integrating core elements of IPS into existing structures rather than completely overhauling them. Grassroots efforts to expand IPS programs and a flexible stance toward quasi-IPS services appear to be key approaches in the Japanese context. Additionally, reframing employment services for individuals with mental illness as a human rights issue, aligned with international standards such as the CRPD, may offer additional leverage for systemic change. Finally, balancing resource allocation between services for people with mild and those with a diagnosis of SMI is a pressing issue that requires thoughtful political discussion. In summary, grassroots activities, advocate activities, incorporation of employer and client perspectives, and balanced discussion between several stakeholders are essential to effectively disseminate IPS in Japan. In addition, this study also found that macro-level research on IPS implementation in Japan remains scarce. Priority areas for future research include assessing employer perspectives, quantifying rejection rates across employment service agencies, and continuously evaluating the impacts of policy and funding reforms on the availability, accessibility, and scale-up of IPS programs.

## Footnotes

1
https://jipsa.jp/

2
https://vfoster.org/nippon-foundation/
