# Supplementary material for: Structural barriers to implementing individual placement and support in Japanese disability policies and Labor systems
Source: Public Health Rev. 2026 Jul 7;47:1608934. doi: 10.3389/phrs.2026.1608934 (PMC13384904; doi:10.3389/phrs.2026.1608934)
Supplement: Supplementary file 1 [file Supplementaryfile1.docx]

**Supplementary Table 1.**

**OECD’s proposed stages for developing integrated mental health, skills, and work policies**

| **Stage** | **Title** | **Key Characteristics** | **Main Policy Priorities** |
| --- | --- | --- | --- |
| **Stage 1** | Developing the right rhetoric | Countries often lack a national mental health plan, or existing plans give little attention to integrated mental health, skills, and employment services. Mental health policy is still at an early stage, and stigma against people with mental health conditions remains widespread. | Expanding community-based mental health service capacity and, in some cases, increasing public awareness of mental health. |
| **Stage 2** | Building the foundations for integrated mental health, skills, and work policy | Countries have national mental health plans that recognize the importance of integrated services, but implementation is limited to pilot projects or small-scale initiatives inside or outside the health system. | Continuing to expand community-based services and raise awareness further, while also laying the groundwork for integrated mental health, skills, and employment policy. |
| **Stage 3** | Shifting from trials to a scaled-up integrated approach | Countries have established strategies for integrated service delivery. Effective and innovative pilot programs are relatively widespread, but they are often not yet scaled up. Public awareness has generally reached a basic level, and community mental health services form the main support base. | Scaling up integrated policies and services, and addressing structural barriers to inter-agency coordination. |
| **Stage 4** | Integrated mental health, skills, and work plans in practice | Countries are implementing well-developed integrated plans through large-scale, evidence-based interventions, although progress may vary across policy areas. Broader performance indicators, including employment-related targets, are increasingly incorporated into national mental health plans. | Further expanding access to integrated services and addressing remaining gaps in support for people with mental health conditions. |

**Supplementary Table 2.**

**Characteristics of the three divisions for employment services and relevant services in Japan**

|  | **Labor Division (Labor bureau)** | **Disability Welfare Division** | **Mental Health and Disability Division** |
| --- | --- | --- | --- |
| **Mission/aim** | Promote measures for the employment of persons with disabilities with the goal of realizing an inclusive society in which persons with disabilities can fully realize their aspirations, use their abilities and aptitudes, and play active roles in ways that accommodate the characteristics of their disabilities, and in which working together with persons with disabilities is considered a natural and ordinary part of society. [1] | Promote disability health and welfare policies, including disability welfare services, with the aims of creating a society in which persons with disabilities can live ordinary lives and participate fully as members of their local communities, and reforming the disability policy system. [2] | Establish a community-based integrated care system responsive to mental health conditions in which all people, regardless of the presence or severity of mental illness, are able to live safely as members of their communities, with comprehensive access to medical care, disability welfare and long-term care services, housing, social participation including employment, mutual community support, and education. [3] |
| **Focus** | Citizens’ work environment | Improvement in the lives of people with disability | Improvement in the citizens’ mental health and the lives of people with mental illness |
| **Laws related to employment for people with mental illness** | Act for Promotion of Employment of Persons with Disabilities | Comprehensive Support Act for Persons with Disabilities | Health insurance law |
| **Employment services and regulation** | - Work and life support centers for persons with disabilities - Job coach service (utilization only after individuals are employed) - Legal employment rate of people with disabilities - Subsidies for companies related to the employment of people with disabilities | - Transition Support for Employment - Sheltered workshop   Continuing employment type A  Continuing employment type B   - Employment retention service | - Psychiatric day-care - Outpatient occupational therapy - Life counseling by social workers |
| **Other features** | - Services are not limited to people with mental illness but are offered to anyone with a disability - Few mental health professionals | - All services are not limited to people with mental illness but are offered to anyone with a disability - Few mental health professionals | - Few employment service professionals |

[1] Ministry of Health, Labour and Welfare. Employment measures for people with disabilities Available from: <https://www.mhlw.go.jp/stf/seisakunitsuite/bunya/koyou_roudou/koyou/shougaishakoyou/index.html>

[2] Ministry of Health, Labour and Welfare. Welfare for people with disabilities Available from: <https://www.mhlw.go.jp/stf/seisakunitsuite/bunya/hukushi_kaigo/shougaishahukushi/index.html>

[3] Ministry of Health, Labour and Welfare. Community-based integrated care system focusing on mental health care. Available from: <https://www.mhlw.go.jp/stf/seisakunitsuite/bunya/chiikihoukatsu.html>

**Supplementary Table 3.**

**Annual number of individuals with disabilities obtaining new employment through public employment security offices**

| **Year** | **Mental illness** | **Physical disability** | **Intellectual disability** | **Total** |
| --- | --- | --- | --- | --- |
| 2000 | 1,614 | 19,244 | 7,414 | 28,272 |
| 2001 | 1,629 | 18,299 | 7,069 | 26,997 |
| 2002 | 1,890 | 19,104 | 7,269 | 28,263 |
| 2003 | 2,493 | 22,011 | 8,249 | 32,753 |
| 2004 | 3,592 | 22,992 | 9,102 | 35,686 |
| 2005 | 4,665 | 23,834 | 10,154 | 38,653 |
| 2006 | 6,739 | 25,490 | 11,441 | 43,670 |
| 2007 | 8,479 | 24,535 | 12,186 | 45,200 |
| 2008 | 9,456 | 22,623 | 11,889 | 43,968 |
| 2009 | 10,929 | 22,172 | 11,440 | 44,541 |
| 2010 | 14,555 | 24,241 | 13,164 | 51,960 |
| 2011 | 18,845 | 24,864 | 14,327 | 58,036 |
| 2012 | 23,861 | 26,573 | 16,030 | 66,464 |
| 2013 | 29,404 | 28,307 | 17,649 | 75,360 |
| 2014 | 34,538 | 28,175 | 18,723 | 81,436 |
| 2015 | 38,396 | 28,003 | 19,958 | 86,357 |
| 2016 | 41,367 | 26,940 | 20,342 | 88,649 |
| 2017 | 45,064 | 26,756 | 20,987 | 92,807 |
| 2018 | 48,040 | 26,814 | 22,234 | 97,088 |
| 2019 | 49,612 | 25,484 | 21,899 | 96,995 |
| 2020 | 40,624 | 20,025 | 19,801 | 80,450 |
| 2021 | 45,885 | 20,829 | 19,957 | 86,671 |
| 2022 | 54,074 | 21,914 | 20,573 | 96,561 |
| 2023 | 60,598 | 22,912 | 22,201 | 105,711 |
| 2024 | 65,518 | 22,704 | 22,449 | 110,671 |
| 2025 | 66,580 | 21,463 | 22,215 | 110,258 |

Ministry of Health, Labour and Welfare. Job Placement for Persons with Disabilities in Fiscal 2025. Press release (2026). Available online at: <https://www.mhlw.go.jp/content/11704000/001713333.pdf> (Accessed June 22, 2026).

**Supplementary Figure 1.**

**Review process of systematic search**

**A) MHLW-funded IPS research reports B) Academic bibliography database**

**Database search (PubMed & Ichushi)**

**77 article records**

**Database search**

**18 projects (23 reports)**

**Excluded:**

- Trial-based study (e.g., RCT) (n= 4)
- Fidelity or process analysis research (n = 5)
- Case study (n = 1)
- Studies focusing on vocational function (n = 3)
- Studies not focusing on employment (n = 5)
- Not Japanese study (n = 2)
- Non-peer reviewed article (n = 55)

**Excluded:**

- Project regarding job retention support (n = 2)
- Project regarding job assessments (n= 3)
- Project regarding sheltered workshops (n = 2)
- Project on an online evidence platform (n = 1)
- Project on rehabilitation for higher brain dysfunction (n = 2)
- Project other than employment services (n = 4)

**IPS and policies**

**2 articles**

**IPS research project**

**4 projects**
